# Dynamics of marsh-mangrove ecotone since the mid-Holocene: A palynological study of mangrove encroachment and sea level rise in the Shark River Estuary, Florida

**DOI:** 10.1371/journal.pone.0173670

**Published:** 2017-03-10

**Authors:** Qiang Yao, Kam-biu Liu

**Affiliations:** Department of Oceanography and Coastal Sciences, School of the Coast and Environment, Louisiana State University, Baton Rouge, Louisiana, United States of America; Centro de Investigacion Cientifica y de Educacion Superior de Ensenada Division de Fisica Aplicada, MEXICO

## Abstract

Sea level rise and the associated inland shift of the marsh-mangrove ecotone in south Florida have raised many scientific and management concerns in recent years. Holocene paleoecological records can provide an important baseline to shed light on the long-term dynamics of vegetation changes across this ecotone in the past, which is needed to predict the future. In this study, we present palynological, X-ray fluorescence, and loss-on ignition data from four sedimentary cores recovered from a 20-km marine-to-freshwater transect along the Shark River Estuary, southwest Everglades, to document the patterns and processes of coastal vegetation changes in response to sea level rise since the mid-Holocene. Our record indicates that freshwater marsh progressively replaced marl prairies at the Shark River Estuary between 5700 and 4400 cal yr BP. As marine transgression continued, marine influence reached the threshold necessary for mangroves to establish at the current mouth of the Shark River Slough at 3800 cal yr BP. During the next 3000 years, although sea level rise in the Western North Atlantic slowed down to 0.4 mm/yr, a spatial and temporal gradient was evident as the marsh-mangrove ecotone shifted inland by 20 km from 3800 to 800 cal yr BP, accompanied by a gradual landward replacement of freshwater marsh by mangrove forest. If sea level continues to rise at 2.33 mm/yr in the 21^st^ century in south Florida, it is possible that marine influence will reach the threshold for mangroves to establish in the central Everglades, and we could expect a much more aggressive mangrove encroachment toward the northern and interior parts of south Florida in the next few centuries.

## Introduction

Contemporary global climate changes are expected to cause many unprecedented ecological impacts on coastal ecosystems around the globe [1]. In North America, one of the most extensive brackish marshes and the largest mangrove swamp is located in the coastal areas of the Everglades in south Florida [2]. More than 200,000 ha of mangrove forests, sawgrass marshes, and sloughs extend along the modern coast from Naples to Florida Bay [3, 4]. These coastal ecosystems are arranged in well-defined compositional zones parallel to the coast, with mixed mangrove stands near the coast giving way to brackish marshes (graminoid-mangrove mixtures), and then freshwater marshes [5, 6]. The brackish marshes serve as a region of transition between mangroves and freshwater marshes, hence the ecotone. Since AD 1930, the marsh-mangrove ecotones have been reported to have moved inland in several coastal areas in the Everglades, accompanied by landward replacement of freshwater marshes by mangrove communities (mangrove encroachment) [6, 7, 8]. Among various factors driving the distribution of mangroves and marshes, sea level rise has been reported to be the primary driver (e.g., inundation and saltwater intrusion) of mangrove encroachment in south Florida [6, 7, 9]. More importantly, if sea level rise continues at the current rate (~2.33 mm/yr) in south Florida [10], the majority of coastal ecosystems in the Everglades will face profound hydrological and structural changes by the end of the 21^st^ century [11, 12]. Studies from around the globe have suggested that ecotones are very dynamic system and sensitive to environmental change [7, 13, 14]. Therefore, monitoring the vegetation dynamics across the marsh-mangrove ecotone is key to understanding the ecological impacts of sea level rise in the Everglades.

Currently, ecological studies in south Florida have monitored the distribution of marshes and mangrove forests for a few decades [6, 7, 8, 15, 16], but paleoecological data on centennial to millennial timescales and along environmental gradients are needed to study the patterns and processes of vegetation dynamics across the ecotone [13, 17]. Unfortunately, major gaps exist in the paleoecological data network. The Holocene paleoecological record can provide us with the best analog to fill this gap, because during this epoch marine transgression has shaped the modern coastal zonation pattern in south Florida [11, 18, 19]. Paleoecological studies have successfully documented the Holocene vegetation dynamics across major upland or continental ecotones in North America [13, 20–23]. However, well dated paleoecological records are rare from coastal zones in the North American tropics and subtropics [24–33], especially the marsh-mangrove ecotone in Florida and the northern Gulf of Mexico coast where mangroves reach their northernmost distributions in continental North America [30, 31]. The existing pollen records from south Florida focus on the effects of climate change on coastal ecosystems at individual sites [22, 30, 31], but data from a transect or network of sites are needed to provide a spatiotemporal perspective on the movements of this marsh-mangrove ecotone during the Holocene in response to postglacial sea level rise.

**Fig 1:**
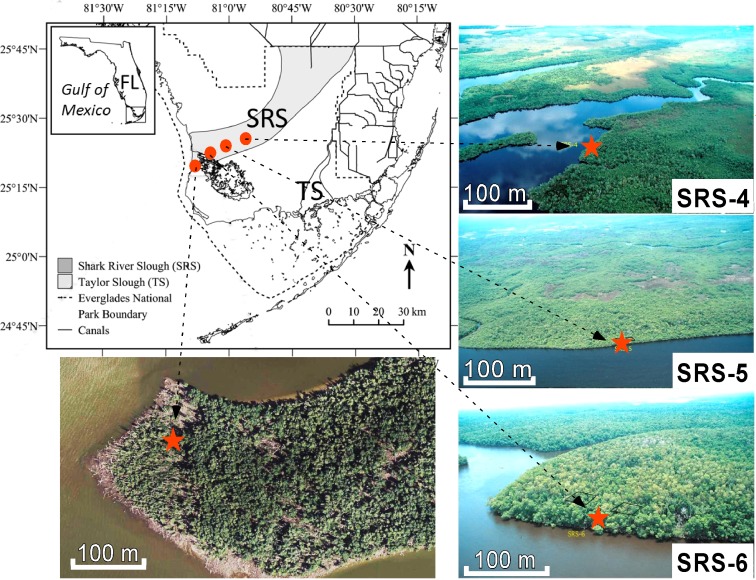
Top left: Location of the Shark River Slough (SRS). **Right**: aerial photos of study sites SRS-4, SRS-5, and SRS-6 (base map and aerial photos adapted from FCE LTER: http://fcelter.fiu.edu/. Copyright permission is acquired). **Bottom left**: satellite photo of site SRM (adapted from USGS National Map Viewer: http://viewer.nationalmap.gov/viewer/). Red stars represent location of the coring sites. Site numbers are tied to core ID described in S1 Table.

In this study, we present palynological, X-ray fluorescence (XRF), and loss-on ignition (LOI) data for four sedimentary cores recovered from a 20 km marine-to-freshwater transect along the estuary of Shark River Slough in southwest Everglades (Fig 1, S1 Table). The proxy record from SRM [30], the most marine-influenced site at the mouth of the Shark River Estuary, suggests that mangroves started to appear at ~4000 cal yr BP as a result of marine transgression. Between 4000 and 3000 cal yr BP, mangroves gradually replaced freshwater mashes, and at ~1150 cal yr BP, a dense *Rhizophora mangle*-dominated mangrove forest was formed at the mouth of the Shark River Estuary [30]. The new proxy records from three more study sites to the east of SRM along a steep salinity gradient provide a unique dataset and spatiotemporal perspective on the Holocene evolution and migration of the marsh-mangrove ecotone. These proxy records along a coastal environmental transect allow us to focus on: (1) the coastal vegetation dynamics in response to the mid- to late-Holocene sea level rise in the Everglades; and (2) the spatiotemporal gradient of mangrove encroachment along the Shark River Estuary. This study will shed light on the future marsh-to-mangrove conversion in south Florida and deepen our understanding of the long-term ecological impacts of sea level rise on coastal ecosystems.

## Materials and methods

### Study sites

This study does not involve human participants, specimens or tissue samples, or vertebrate animals, embryos or tissues. Permission for field activities is granted by Florida Bay Interagency Science Center-Everglades National Park (FBISC-ENP). The four coring sites are placed to follow a steep salinity gradient along the estuary of Shark River Slough, the largest freshwater outlet in the Everglades [2]. Site SRM is situated in a fringing mangrove forest at the mouth of the Shark River Slough [30], and sites SRS-6, SRS-5, and SRS-4 are located at approximately 4 km, 8 km, and 20 km upstream from the mouth, respectively. The hydroperiods in sites SRM, SRS-6, and SRS-5 are influenced mainly by tidal cycles, whereas that in site SRS-4 is controlled mainly by freshwater discharge. Accordingly, soil pore-water salinity decreases significantly upstream from ~30 ppt at SRM to 4.6 ± 1.1 ppt at SRS-4, and SRM is flooded by tides twice as often as at SRS-4 (S1 Table) [34].

Currently, three true mangrove species (*Rhizophora mangle*, *Laguncularia racemosa*, and *Avicennia germinans*) and a mangrove associate (*Conocarpus erectus*) are found in the Everglades. At site SRM, *Laguncularia* and *Rhizophora* are co-dominant species [33, 34, 35]. Site SRS-6 has the highest plant diversity along the Shark River transect, whereas *Laguncularia*, *Rhizophora*, and *Avicennia germinans* are co-dominant species [30]. Site SRS-5 is totally occupied by *Rhizophora* with very few other mangrove species. Site SRS-4 is occupied by *Rhizophora* with *Laguncularia* as co-dominant species. It is the only site along the transect where *Conocarpus erectus* is found and *Avicennia* is absent.

A 525, 445, 250, and 185 cm sediment core was extracted from site SRM, SRS-6, SRS-5, and SRS-4, respectively by using a Russian peat borer in May 2010. All cores retrieved contain the complete depositional history above the bedrock platform. The 50-cm core segments were measured and photographed in the field before being wrapped by layers of plastic wrap and duct-tapes to ensure minimal water loss. More information about the vegetation, hydroperiod, and GPS location of each site are described in online supplementary content (S1 Table).

### Chronology

Thirty-four samples consisting of leaf fragments and pieces of wood were selected under a dissecting microscope and sent to the NOSAMS Laboratory at Woods Hole Oceanographic Institution (WHOI) and Beta Analytic Inc. for AMS ^14^C measurements. Among these, 14 were from core SRM, 7 were from core SRS-6, 6 were from core SRS-5, and 7 were from core SRS-4. All ^14^C dates were calibrated using the Calib 7.0 program [36] and reported as calibrated years before present (cal yr BP) in this study. The chronology of core SRM was developed by using BACON version 2.2 [37] and has been previously established [30]. Core SRS-6, SRS-5, and SRS-4 each contained fewer dates, therefore the sedimentation rates for these 3 cores were determined by linear interpolation between the median of corresponding calibrated range (2-σ). Two ^14^C dates each were rejected from core SRS-6, SRS-5, and SRS-4 due to extreme age reversal. More information about the ^14^C samples is described in online supplementary content (S2 Table).

### Pollen, XRF, and LOI

**Fig 2:**
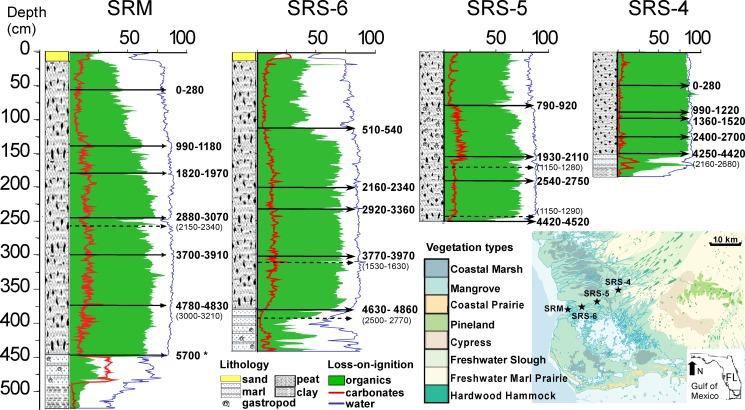
Lithology, loss-on-ignition diagram, and ^14^C dates for core SRM, SRS-6, SRS-5 and SRS-4. Arrows with solid lines point to accepted dates at corresponding depth. Arrows with dash lines point to rejected dates at corresponding depth. A map showing vegetation types surrounding the study sites is added to the figure to facilitate the interpretation of data.

**Fig 3:**
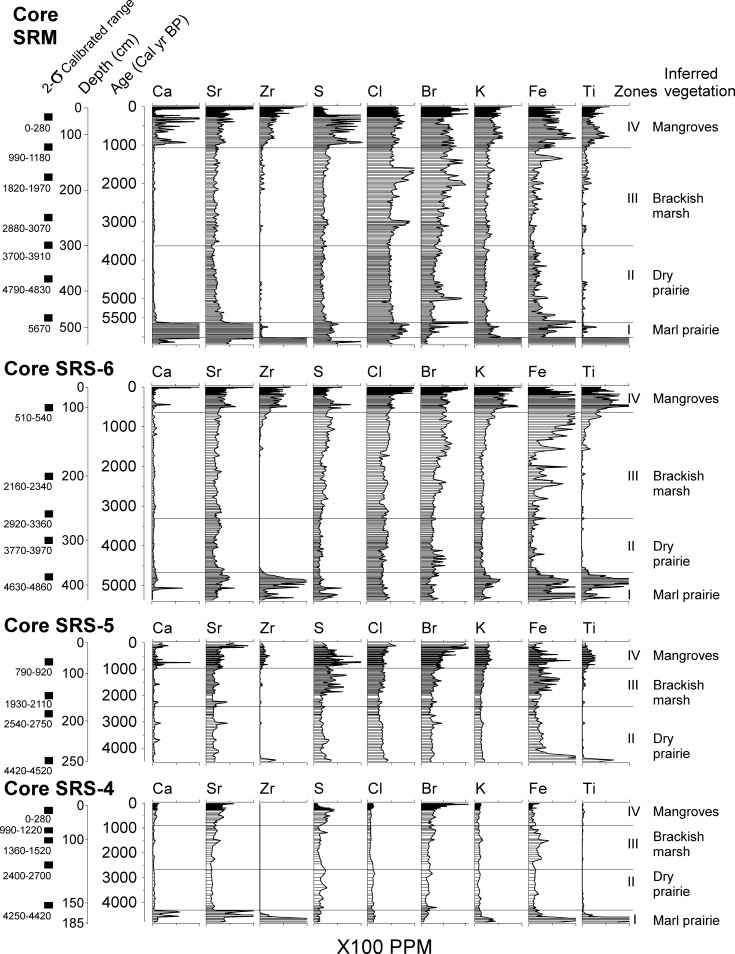
XRF diagrams from core SRM, SRS-6, SRS-5, and SRS-4 plotted against calibrated age (cal yr BP). The 2-σ calibrated range of accepted ^14^C dates are listed at the left side of each core at corresponding depths.

**Fig 4:**
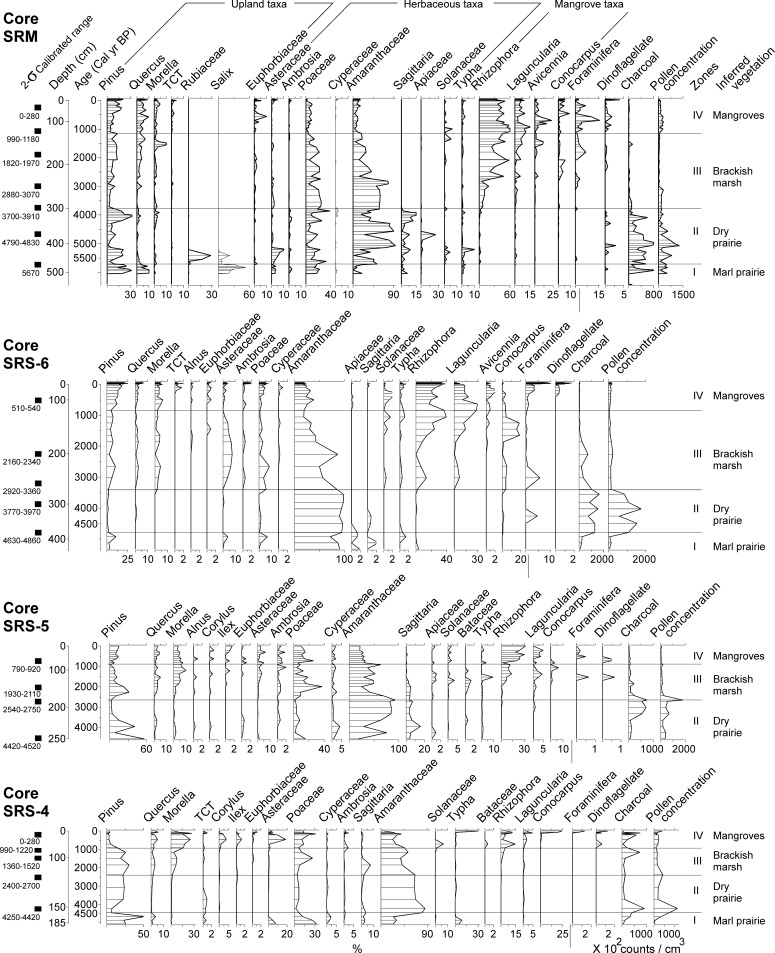
Pollen percentage diagrams for core SRM, SRS-6, SRS-5, and SRS-4 plotted against calibrated age (cal yr BP). The 2-σ calibrated range of accepted ^14^C dates are listed at the left side of each core at corresponding depths. The concentration curves for marine planktons, charcoal fragments, and total pollen are added on the right to facilitate comparison.

**Fig 5:**
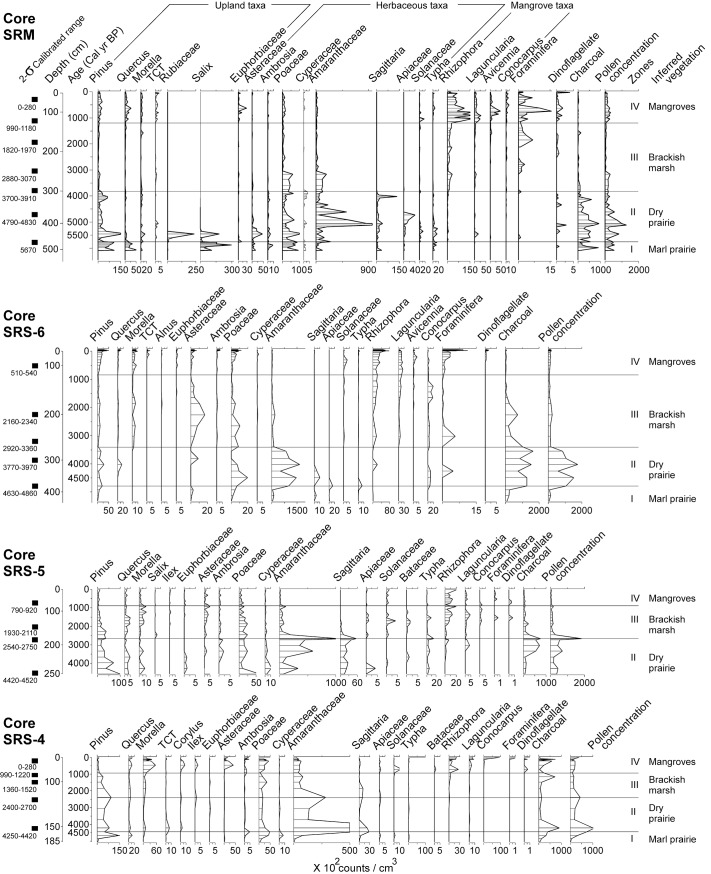
Concentration diagrams of pollen, marine planktons, and charcoal fragments for core SRM, SRS-6, SRS-5, and SRS-4 plotted against calibrated age (cal yr BP). The 2-σ calibrated range of accepted ^14^C dates are listed at the left side of each core at corresponding depths.

Laboratory procedures for pollen, XRF, and LOI analyses followed those described in Yao et al. [30]. XRF analysis was performed at 2 cm interval on all cores to measure elemental concentrations (ppm) of major chemical elements [30, 38]. XRF analysis has been successfully used to identify chemical elements that are primarily derived from marine (e.g., Ca, Sr, and Zr) and terrestrial (e.g., Fe, Ti, and K) environments in coastal environments [30, 38, 39]. LOI analysis was performed at 1 cm interval to establish sediment stratigraphy [40]. The LOI and XRF results of all cores are displayed in Figs 2 and 3. For palynological analysis, 67 samples were taken from core SRM at 5 to 10 cm intervals; 39 samples were taken from core SRS-6 at 10 to 15 cm intervals; 26 samples were taken from core SRS-5 at 10 cm interval; and 20 samples were taken from core SRS-4 at 5 to 10 cm intervals. Approximately 300 grains of pollen and spores, including both terrestrial and aquatic taxa, were counted in most samples and used as the pollen sum for the calculation of pollen percentages. Foraminifera linings, dinoflagellates, and charcoal fragments (>10 μm in size) were also counted for each sample, but their percentages were calculated outside the sum. The identification of pollen was based on Willard et al. [41, 42]. The palynological results are reported in percentage (%) and concentration diagrams (Figs 4 and 5). Key features of each pollen zone and more detailed description of each core is presented in online supplementary content (S1 Table).

## Results

### Stratigraphy

The LOI and ^14^C dating results of all four cores show a consistent and parallel depositional history during the late-Holocene (Fig 2). Approximately 10 cm of carbonate-rich clastic sediments were found at the top of cores SRM and SRS-6 (Fig 2, S1A Fig), the two most-seaward sites along the transect. The sand layer at core SRM has been previously described and attributed to Hurricane Wilma, a category 3 storm that made landfall near the study site in 2005 [30, 34, 35]. All cores from the Shark River transect primarily consisted of peat (Fig 2). In SRM, the longest core retrieved from the mouth of the River, the peat started to accumulate from ~5700 cal yr BP but it did not start until ~4800 cal yr BP at site SRS-6. The basal ages of the peat sections progressively became younger toward more upstream sites (~4500 cal yr BP at site SRS-5, ~4400 cal yr BP at site SRS-4), and the cores also became shorter upstream. Stratigraphically, the peat sections of all cores were very similar in that they contained high contents of water and organic matter and relatively low percentages of carbonates (Fig 2). Underlying the peat sections in cores SRM, SRS-6, and SRS-4 are 15 to 75 cm of marl sediments that were low in organics and rich in carbonates. Shell hashes of the freshwater snail *Helisoma trivolvis* sp. were present in the marl sediments of cores SRM and SRS-6. At the bottom of cores SRM and SRS-4 were 40 cm and 10 cm of homogeneous basal clay with very low contents of water, organics, and carbonate. Both clay sections were pollen-barren and most likely a bedrock substrate caused by acidic leaching (Fig 2).

### Pollen and XRF results

The XRF, pollen percentage, and pollen concentration results of the four cores are displayed in Figs 3, 4, and 5. Based on the consistence in pollen and XRF results, all cores except SRS-5 are divided into 4 corresponding pollen zones. Core SRS-5 is divided into 3 corresponding pollen zones with Zone I, in the marl sediments, missing at the basal part of the core. The pollen assemblages in each core show similar transitions with the zone boundaries progressively becoming younger upstream from the most marine-influenced towards the more inland sites. The key features of each pollen zone are described in the following paragraphs, and additional information about the pollen diagrams are outlined in S1 Table.

#### Zone I

The oldest pollen assemblage from the Shark River transect appeared in the marl sediments (485 to 450 cm) overlying the basal clay at the bottom of core SRM, which were formed prior to 5700 cal yr BP. This zone is characterized by abundant arboreal taxa (10–30%), particularly *Salix*, *Pinus*, and *Quercus*. Herbaceous taxa (Poaceae, Amaranthaceae, *Sagittaria*, and *Typha*) (5–15%) are also consistently present in this zone. Moving further inland, similar pollen assemblages were deposited in the marl sediments in core SRS-6 (450 to 380 cm) and SRS-4 (175 to 160 cm) prior to 4800 and 4400 cal yr BP, respectively, with *Pinus*, Poaceae, and Amaranthaceae being the most abundant species. Charcoal is becoming more abundant toward the top part of Zone I in all cores. XRF analysis yielded very similar results from the marl sediments in cores SRM, SRS-6, and SRS-4. They generally have high concentrations of Ca, Sr, Fe, and Ti.

#### Zone II

As peat started to accumulate, the pollen assemblage of Zone II in core SRM (450 to 300 cm, 5700 to 3800 cal yr BP) is marked by a distinct shift to herbaceous taxa and abundant charcoal. While the bottom of Zone II in core SRM still contains a mixture of arboreal (*Salix*, *Pinus*, Rubiaceae), and herbaceous taxa (Asteraceae, Poaceae, *Typha*), Amaranthaceae (> 50%) becomes the most dominant pollen taxa from 410 to 350 cm. At the top of this zone *Pinus* (30%) and *Sagittaria* (15%) are the most abundant taxa. At more inland sites, the abundance of charcoal and Amaranthaceae pollen also progressively increases in Zone-II of core SRS-6 (380 to 260 cm, 4800 to 3430 cal yr BP), SRS-5 (250 to 175 cm, 4500 to 2380 cal yr BP), and SRS-4 (160 to 120 cm, 4400 to 2330 cal yr BP). Similar to core SRM, *Pinus*, Poaceae, and *Sagittaria* are consistently present in Zone II of these three cores. XRF analysis shows low concentrations of all detected chemical elements in Zone-II of all cores.

#### Zone III

Zone III is characterized by a major increase of mangrove pollen and decrease of charcoal fragments. From ~3800 cal yr BP, *Rhizophora* pollen started to appear in the pollen assemblage of core SRM, and progressively appeared in core SRS-6 (~3430 cal yr BP), SRS-5 (~2380 cal yr BP), and SRS-4 (~2330 cal yr BP) towards more inland sites. Although *Pinus*, Poaceae, and Amaranthaceae pollen are still very frequent (> 10%) in Zone III of all cores, the abundance of mangrove taxa, especially *Rhizophora*, consistently increase toward the top of this zone in all cores. *Laguncularia* and *Conocarpus* become more abundance at the top of Zone III in core SRM, SRS-6, and SRS-5. *Avicennia* also starts to appear at the top of Zone III in core SRM and SRS-6, but is absent further upstream in cores SRS-5 and SRS-4. Other common taxa include *Quercus*, *Morella*, and Asteraceae. XRF results show higher contents of Br and S toward the top of Zone III in all cores.

#### Zone IV

This zone is characterized by the dominance of mangrove taxa in the pollen assemblage. In Zone IV of core SRM (140 to 10 cm, 1140 cal yr BP to present), *Rhizophora* accounts for >50% of the pollen sum and has very high pollen concentration values. *Rhizophora* pollen are also very abundant in Zone IV of core SRS-6 (130 to 10 cm, 1060 cal yr BP to present), SRS-5 (80 to 0 cm, 860 cal yr BP to present), and SRS-4 (80 to 0 cm, 860 cal yr BP to present), even though its maximum percentages were decreasing from ~50% at SRM to ~15% at SRS-4 as a function of the diminishing marine influence upstream. At the top 25 cm of core SRS-4, *Conocarpus* becomes the most dominant pollen taxon. Though less abundant, the percentage and concentration curves for *Laguncularia* generally mimic those for *Rhizophora* in all four cores. *Avicennia* starts to appear towards the top of Zone III and reaches a peak in the bottom half of Zone IV in core SRM and SRS-6, but it is absent in SRS-5 and SRS-4 upstream. While the pollen percentages of *Rhizophora* are increasing in towards the core top, those of Amaranthaceae show an opposite trend, decreasing sharply in Zone III and Zone IV in all four cores. Other herbaceous taxa (Asteraceae, *Ambrosia*, and Poaceae) continue to be present in Zone IV in all four cores, but they seem to be somewhat more frequent in the fresher sites (SRS-5, SRS-4) than in the more marine-influenced sites (SRS-6, SRM). Among the arboreal taxa, *Pinus* percentages increase slightly towards the top of cores SRM, SRS-6, and SRS-5 but decrease sharply in Zone IV of the most inland site (SRS-4). *Morella*, however, shows the opposite trend and increases dramatically in Zone IV as *Pinus* decreases significantly at the latter site. In addition, foraminifera linings and dinoflagellates are present in higher abundance and the concentration of charcoal remains low in Zone IV of all cores than in the underlying zones. XRF results show a significant increase in the concentrations of most chemical elements (especially Ca, Sr, Zr, and Br) in Zone IV of all cores.

## Discussion

### Holocene environmental evolution of Shark River Estuary

**Fig 6:**
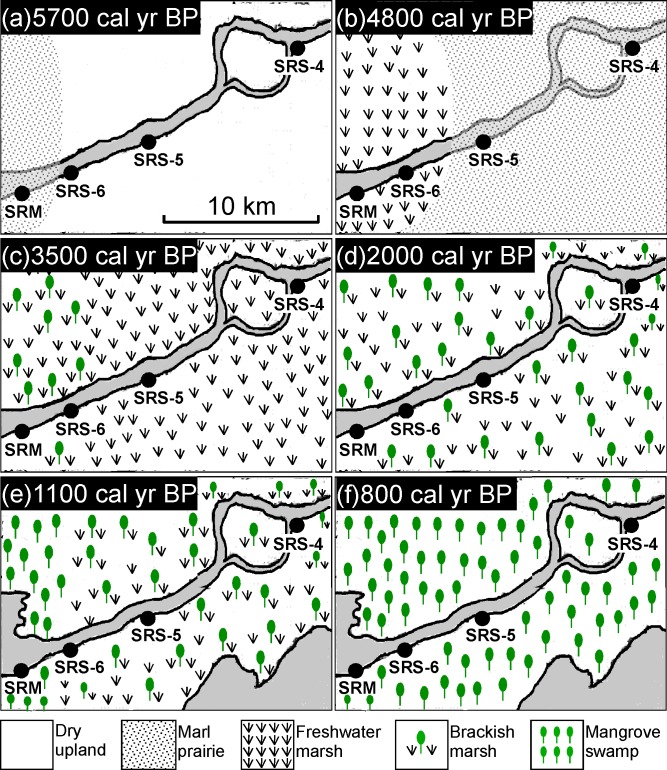
Model of the evolution of the Shark River Estuary. The time horizon for each figure is selected to show each stage of the development of the Shark River Estuary.

To provide a regional perspective, the four pollen records from the Shark River transect are correlated schematically, and each stage of the landscape-scale vegetation pattern is displayed in Fig 6. Where the study area is situated today appears to be a dry and upland environment prior to the mid-Holocene, ca. 6000 yr BP. Basal clay layers in core SRM and SRS-4 are devoid of pollen. This suggests a subaerial condition below the “wetness” threshold for pollen preservation. The absence of pollen also precludes knowledge of the vegetation types present. Our study sites were most likely situated on an arid or frequently dried out terrestrial environment that was not inundated long enough to promote marl or peat accumulation during the early to mid-Holocene. Some time prior to 5700 cal yr BP, marl sediments rich in shell hashes of freshwater snails had started to accumulate at the current mouth of the Shark River Slough (site SRM) and continued to spread upstream (Fig 6A). By 4800 cal yr BP, marl prairies had already occurred at site SRS-4, at least 20 km from the mouth (Fig 6B). Abundant *Pinus*, *Salix* and Amaranthaceae in the pollen assemblage suggest that the vegetation of the marl prairies contained a mixture of short-hydroperiod and longer-hydroperiod prairies (Figs 4 and 5), perhaps also with pine savannas in the immediate vicinity. The spreading of marl prairies toward more inland areas over subaerial substrate suggests a gradual rise in the phreatic aquifer [19, 43]. Similar marl prairies are present in the interior of the Everglades today (e.g., Big Cypress National Preserve and Long Pine Key) [44]. These habitats currently have hydroperiods lasting less than 12 months and are thus dry seasonally, tending to burn more than once a decade, and even every 1–2 years if located adjacent to pine savannas [44–46]. Such high fire frequency could be inferred from the abundance of charcoal particles in pollen Zone I of core SRM, SRS-6, and SRS-4. Most importantly, our study not only indicates that marl prairies have been present in southwestern Florida since the mid-Holocene, but it also implies that early wetland landscapes in the Shark River Estuary were likely to have resembled the fire-maintained landscapes occurring today in the interior parts of the Everglades [44, 46].

Peat accumulating landscapes started to appear at site SRM at ~5700 cal year BP, suggesting the development of freshwater marshes (Fig 6A). The fresh marsh landscape had spread to site SRS-6 by 4800 cal yr BP, and it dominated the entire study area from 4400 to 3800 cal yr BP (Figs 4 and 5). Abundant Amaranthaceae and charcoal fragments suggest that this stage of the wetland development (pollen Zone II) is characterized by a landward replacement of upland taxa by herbaceous taxa (Figs 4 and 5). The consistently low values of all elements in Zone II suggest that it was a freshwater environment with little to no marine influence [30]. These pollen and XRF data are consistent with peat-accumulating freshwater marsh similar to the ones existing in present-day northern to central Everglades [43]. These habitats today have 12 month hydroperiods. The increase of Amaranthaceae and charcoal particle in these freshwater wetland habitats has been attributed to natural lightning-ignited fires [46] and hurricanes [47], when open space is generated and colonized by species like *Amaranthus australis* [44]. Such transition from marl prairies to peat-accumulating freshwater marsh suggests an extended hydroperiod due to elevated water table between 5700 and 3800 cal yr BP. Although marine influence was minimal during this period, this time-transgressive rising of water table toward inland was a clear evidence of marine transgression because the Shark River Slough lies atop the permeable Tamiami limestone where the water table was very sensitive to sea level fluctuations [19],

With the sea level in south Florida continuing to rise after the mid-Holocene [19, 48], freshwater environment at the Shark River Estuary was progressively replaced by brackish marsh after 3800 cal yr BP (Fig 6C and 6D). This transition is clearly recorded by the pollen and XRF data (Figs 4 and 5). Linings of foraminifera started to appear with greater regularity during this period (pollen Zone III), while mangrove species started to appear at site SRM and gradually expanded toward site SRS-4. Additionally, the abundance of microscopic charcoal fragments diminished, and concentration of Br and S increased. These evidences suggest that our study area was experiencing fewer fires and certainly under greater marine influence. Today, similar brackish marsh occurs as an ecotone between mangrove forest and freshwater marsh in the Everglades [43]. Using this as a modern analog, it could be inferred that around 3800cal yr BP, site SRM was occupied by grasses and *Amaranthus*, with some scrub *Rhizophora* and occasional *Avicennia*, *Laguncularia*, and *Conocarpus*. Between 3800 and 2300 cal yr BP, with more mangrove species colonizing upstream toward site SRS-4, the marsh-mangrove ecotone expanded 20 km toward inland. The entire study area was occupied by such graminoid-mangrove mixtures until ~1100 cal yr BP (Fig 6D).

The period from ~1100 to 800 cal yr BP is characterized by the formation of the modern shoreline (Fig 6E and 6F). By 1100 cal yr BP, Zr, the main constituent for Florida beach sand [49], started to consistently appear at site SRM, SRS-6, and SRS-5 (Fig 4). Concurrently, concentration of Ca, Sr, and Br increased significantly at all 4 study sites (Fig 3). Higher content of Zr, Ca, Sr, and Br has been described as a direct indicator of marine sediments [30]. Because tides and episodic storms are the only mechanisms that can consistently bring in marine sediments, this chemical signature suggests that our study area started to receive sand through tidal exchange and hurricanes at that time. Most importantly, the concentrations of all mangrove species (especially *Rhizophora*) and marine microfossils (foraminifera and dinoflagellate) increased significantly by 1100 cal yr BP at site SRM (Fig 5). These results suggest that a mixed mangrove forest (with all three mangrove species and *Conocarpus*) similar to today’s was established at the mouth of the Shark River Slough. Similar mixed mangrove forest was established at site SRS-6 at ~1000 cal yr BP, and spread to inland sites (SRS-5 and SRS-4) at ~ 800 cal yr BP (Fig 6E and 6F). Hence, transition to the modern coastal mangrove ecosystem was completed by about 800 cal yr BP in the Shark River Estuary. Nevertheless, *Conocarpus*, a mangrove associate typically growing in upland environment, outcompeted *Rhizophora* and became the dominated species at our furthest upstream site SRS-4 since 100 cal yr BP (Figs 4 and 5). Site SRS-4 is located 20 km from the coast and experiences very little tidal influence today [35]. The abrupt increase of *Conocarpus* at site SRS-4 might be associated with significant reduction of surface and groundwater flows throughout the Everglades since the early 20^th^ century due to human activities [50].

### Spatiotemporal gradient of mangrove encroachment into freshwater marsh

Our pollen records indicate that the development of various vegetation regimes was not synchronous throughout the Shark River Estuary. Instead, a spatial and temporal gradient was evident in the vegetation changes as the marsh-mangrove ecotone continually moved further inland and upstream under the influence of regional sea level rise (Fig 6). With rapid sea level rise (2.3 mm/yr) elevating the Florida aquifer during the mid-Holocene [11, 18, 48, 51], freshwater marsh species first appeared at site SRM at ~5700 cal yr BP, and reached site SRS-4 at 4400 cal yr BP. During the next 600 years, as marine transgression continued under rapid sea level rise in south Florida, geomorphological conditions became favorable for mangroves to establish in the study area [30, 31]. At 3800 cal yr BP, mangroves started to appear at site SRM, and then colonized site SRS-6 at 3500 cal yr BP, resulting in the formation of brackish marsh (ecotone between freshwater marsh and mangroves). After 3000 cal yr BP, although the rate of sea level rise declined to 0.4 mm/yr [18, 48], the ecotone continued to move inland during the next 2200 years, accompanied by a gradual landward replacement of freshwater marsh by mangrove swamp.

In addition to sea level rise, drivers behind the mangrove encroachment in coastal regions of North America include rise in winter air temperature [15, 16], shortage in freshwater input [52], and natural disturbances [53]. In areas below 25°N where winters are warm, winter temperature should not be a limiting factor for mangrove survival or to influence the balance between marsh and mangrove communities in south Florida [54]. Freshwater input should be abundant in our study area as the Shark River Slough is the largest freshwater outlet in the Everglades [2]. In addition, there is no evidence of any natural disturbances (e.g., fire and hurricanes) operating at a scale that can alter the coastal vegetation zonation pattern during the late-Holocene in south Florida. Accordingly, our record suggests that once the marine influence reached the threshold necessary for mangroves to colonize the mouth of the Shark River Slough, under a stable climate, the marsh-mangrove ecotone continued to shift landward at a steady pace even though regional sea level rise slowed down to 0.4 mm/yr during the late-Holocene [48]. As evident from the pollen results, the marsh-to-mangrove conversion was a dynamic and time-transgressive process (Fig 6). From the colonization of mangroves at site SRM, till the establishment of mangrove forest at site SRS-4, the ecotone shifted 20 km landward and upstream in roughly 3000 years (3800 to 800 cal yr BP), or an estimated rate of about 7 km per millennium.

In contrast, the marsh-mangrove ecotone has shifted inland by as much as 3.3 km in areas north of Florida Bay and Biscayne Bay in the southeast Everglades since AD 1950 [6], and significant mangrove encroachment into brackish marsh has been documented at the north and east side of the Shark River Slough since AD 1930 [7, 8]. These recent mangrove encroachments are much more aggressive than that occurring in our study area during the late-Holocene. The significantly faster inland shift of the ecotone can probably be attributed to the rapid sea level rise in south Florida between AD 1930 and 1990 (3–4 mm/yr) [48], compared with 0.4 mm/yr during the late-Holocene [48]. Currently, sea level rise (2.33 mm/yr) in south Florida is approximately the same rate as that occurring prior to 3800 cal yr BP (2.3 mm/yr) [10, 48], a time when marine influence reached the threshold for mangroves to establish in the coastal Everglades. In addition, since anthropogenic activities (agriculture and urbanization) have dramatically reduced the seasonality of freshwater flow and affected the surface and groundwater flows throughout the Everglades [50, 55], the hydrological and salinity condition is more favorable for mangroves to migrate landward [6, 7]. If sea level rise continues at 2.33 mm/yr over a very gently sloping landscape (3 cm/km) and permeable limestone platform in south Florida [2], using our record as an analogue, it is possible to infer that mangroves will colonize the northern and interior parts of the Everglades within the next few centuries. Once the threshold for mangrove establishment is reached, marsh-mangrove ecotone will continue to move inland and upstream and mangrove forest will eventually replace the freshwater marsh and marl prairies in the central Everglades (e.g., Big Cypress National Preserve). We should expect a much more aggressive mangrove encroachment in most coastal areas and even interior parts of the Everglades in the near future. Future studies should closely monitor the stability of ecotones and associated changes in ecosystem resilience, food web dynamics, nutrient cycling, carbon sequestration, and community composition across the Everglades.

The paleoecological record from the Shark River Estuary is consistent with other records from the region. A study from Laguna de la Leche in Cuba shows similar mangrove encroachment after 5000 cal yr BP [32]. Studies from Tampa Bay [56] and Charlotte Harbor [38] also document a transition from freshwater to marine environment after 3500 cal yr BP. In addition, a study from Fakahatchee Strand State Preserve Park, southwest Florida, records an intensification of El Niño-Southern Oscillation (ENSO) after the mid-to late-Holocene [57]. Intensification of ENSO is likely to have increased winter rainfall in the southeastern United States and reduced fire frequency and fire extent across long-hydroperiod prairie landscape [52]. Such increases may facilitate the transition from marl prairie to freshwater marsh in the Everglades as well.

## Conclusions

Pollen, LOI, and XRF records from this study documented the Holocene evolution and migration of the marsh-mangrove ecotone at the Shark River Estuary. Our record indicates that marl prairies were the dominant vegetation type before they were progressively replaced by freshwater marshes after ∼5700 cal yr BP. From 5700 to 3800 cal yr BP, geomorphological conditions became favorable for freshwater marshes and eventually mangrove forests to establish in the study area due to rapid sea level rise. At 3800 cal yr BP, marine influence reached the threshold necessary for mangroves to establish at site SRM. During the next 3000 years, although sea level rise in south Florida slowed down, a spatial and temporal gradient was evident as the marsh-mangrove ecotone continually moved inland and upstream, accompanied by a gradual landward replacement of freshwater marsh by mangrove swamp. From the colonization of mangroves at site SRM, to the establishment of mangrove forest at site SRS-4, the ecotone shifted inland by 20 km in 3000 years (3800 to 800 cal yr BP). This study has provided the most comprehensive paleoecological datasets on the dynamics of coastal vegetation communities under the scenario of sea level rise during the middle to late Holocene. This study also produced the most accurate chronology to date from coastal wetlands in Florida. If sea level continues to rise at the current rate (2.33 mm/yr) in south Florida, our data imply that we should expect a much more aggressive mangrove encroachment in the Everglades in the near future. Such information is critical for the success of the 17.6 billion-dollar Comprehensive Everglades Restoration Plan (CERP) [58].

## Supporting information

S1 Fig a) Photo of storm deposits from the top of core SRM. b) Photo of marl sediments from the bottom of core SRS-6. c) Photo of freshwater snails *Helisoma trivolvis* sp.
(TIF)

S1 Table Key features of the pollen results of each core and description of the study sites. Latitudes and longitudes for study sites are determined using global positioning systems. Composition of the above ground vegetation is based on field observation and previous studies [34, 35]. Up arrow (↑) represents increase in pollen percentage. Down arrow (↓) represents decrease in pollen percentage.
(PDF)

S2 Table Radiocarbon dating results for core SRM, SRS-6, SRS-5, and SRS-4. Dates in parentheses are rejected due to extreme age reversal.
(PDF)
