# Supplementary figures and images for: Dynamics of marsh-mangrove ecotone since the mid-Holocene: A palynological study of mangrove encroachment and sea level rise in the Shark River Estuary, Florida
Source: PLoS One. 2017 Mar 10;12(3):e0173670. doi: 10.1371/journal.pone.0173670 (PMC5345865; doi:10.1371/journal.pone.0173670)

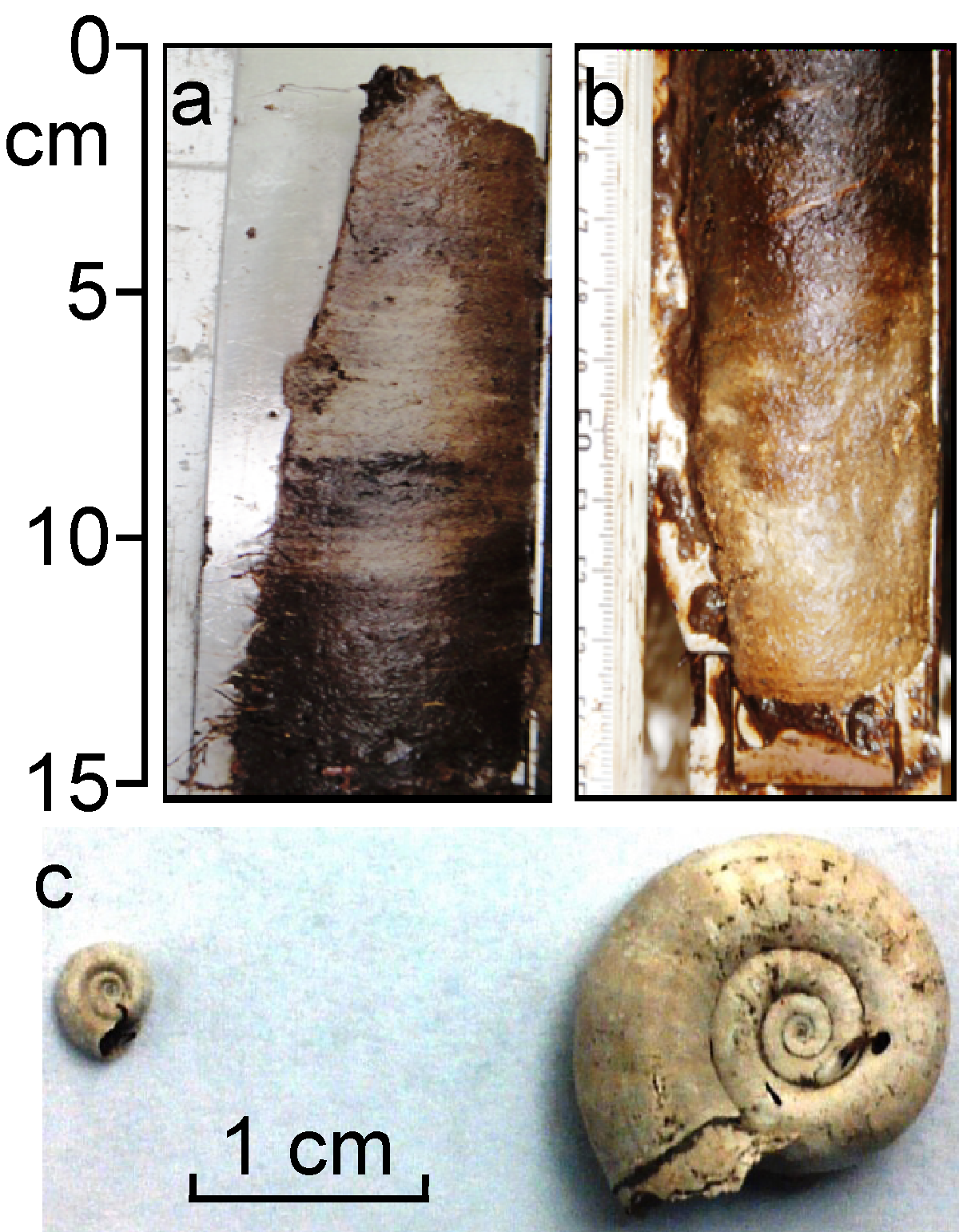

Supplement: S1 Fig — a) Photo of storm deposits from the top of core SRM. b) Photo of marl sediments from the bottom of core SRS-6. c) Photo of freshwater snails Helisoma trivolvis sp. (TIF) [file pone.0173670.s001.tif]
