# Supplementary material for: Dynamics of marsh-mangrove ecotone since the mid-Holocene: A palynological study of mangrove encroachment and sea level rise in the Shark River Estuary, Florida
Source: PLoS One. 2017 Mar 10;12(3):e0173670. doi: 10.1371/journal.pone.0173670 (PMC5345865; doi:10.1371/journal.pone.0173670)
Supplement: S1 Table — Latitudes and longitudes for study sites are determined using global positioning systems. Composition of the above ground vegetation is based on field observation and previous studies [34, 35]. Up arrow (↑) represents increase in pollen percentage. Down arrow (↓) represents decrease in pollen percentage. (PDF) [file pone.0173670.s002.pdf]

Core ID	Current above ground vegetation composition	Coring site information	Pollen zone (cal yr BP)	Modern analog	Pollen signature (%)
**SRM**	Heavily deforested by Hurricane, *Avicennia* fringe, co-dominated by *Rhizophora* and *Laguncularia*, no *Conocarpus*	**Core depth:** 525 cm	I: > 5700	Marl prairie	*Pinus* 20-30%, *Quercus* 5-10%, *Salix* >30%, Poaceae 15-20%
		**GPS:** 25°21'10", -81°06'52"	II: 5700-3800	Freshwater marsh	↑: Amaranthaceae >50%, *Sagittaria* 5-15%, Pinus 10-30%, Rubiaceae 0-30%, Poaceae 5-20%,
		**Pore-water salinity:** ~30 ppt	III: 3800-1140	Brackish marsh	↑: Poaceae >15%, *Rhizophora* 0-50%<br>↓: Amaranthaceae 35-75%
		**Hydroperiod:** ~330 days	IV: 1140-present	Mangroves	↑: *Rhizophora* >50%, *Laguncularia* 5-15%, *Avicennia* 5-25%, *Conocarpus*>5%<br>↓: Amaranthaceae 5-15%, Poaceae <15%
**SRS-6**	*Laguncularia* > *Rhizophora* > *Avicennia*, No *Conocarpus*,	**Core depth:** 445 cm	I: >4800	Marl prairie	Amaranthaceae >80%, Poaceae 0-10%
		**GPS:** 25°21'53", -81°04'41"	II: 4800-3430	Freshwater marsh	↑: Amaranthaceae >90%<br>↓: *Sagittaria* 0-2%, *Pinus* 0-2%, Poaceae 0-2%
		**Pore-water salinity:** 27 ± 2.6 ppt	III: 3430-1060	Brackish marsh	↑: Mangroves, *Pinus* >10%, *Morella* 0-5%, Asteraceae 5-10%, Poaceae 5-10%<br>↓: Amaranthaceae 30-75%, *Sagittaria* disappear
		**Hydroperiod:** 233 days	IV: 1060-present	Mangroves	↑: *Rhizophora* >20%, *Laguncularia* 5-30%, Pinus >15%<br>↓: Amaranthaceae <30%
**SRS-5**	Dominated by tall *Rhizophora*, few *Laguncularia* and *Avicennia*, No *Conocarpus*	**Core depth:** 250 cm	II: 4500-2380	Freshwater marsh	Amaranthaceae >50%, *Sagittaria* 5-15%, *Pinus* 10-60%, Poaceae >10%, Cyperaceae 2-5%
		**GPS:** 25°22'37", -81°01'57"	III: 2380-860	Brackish marsh	↑: Mangroves, *Pinus* >15%, Poaceae 15-30%, *Quercus* >5%, Morella >5%, *Asteraceae* >3%<br>↓: Amaranthaceae 30-50%, *Sagittaria* 0-5%
		**Pore-water salinity:** 20.8 ± 3.1 ppt	IV: 860-present	Mangroves	↑: *Rhizophora* >20%, *Laguncularia* 0-5%<br>↓: Amaranthaceae <25%
		**Hydroperiod:** 197 days			
**SRS-4**	Dominated by scrub *Rhizophora*, *Laguncularia* > *Conocarpus*, no *Avicennia*	**Core depth:** 185 cm	I: >4400	Marl prairie	*Pinus* >50%, Poaceae >15%, Amaranthaceae >30%
		**GPS:** 25°24'34", -80°57'51"	II: 4400-2330	Freshwater marsh	↑: Amaranthaceae >50%<br>↓: *Pinus* 10-25%, Poaceae <15%,
		**Pore-water salinity:** 4.6 ± 1.1 ppt	III: 2330-860	Brackish marsh	↑: Mangroves, *Pinus* >20%, Poaceae >15%<br>↓: Amaranthaceae 40-50%,
		**Hydroperiod:** 165 days	IV: 860-present	Mangroves	↑: *Rhizophora* >10%, *Laguncularia* 2-5%, *Conocarpus* 0-25%, *Morella* >15%, Poaceae >20%<br>↓: Amaranthaceae 30-40%

Table S1. Key features of the pollen results of each core and description of the study sites. Latitudes and longitudes for sites are determined using global positioning systems. Composition of the above ground vegetation is based on field observation and previous studies [32, 35]. Up arrow (↑) represents increase in pollen percentage. Down arrow (↓) represents decrease in pollen percentage.
