# Supplementary material for: Dynamics of marsh-mangrove ecotone since the mid-Holocene: A palynological study of mangrove encroachment and sea level rise in the Shark River Estuary, Florida
Source: PLoS One. 2017 Mar 10;12(3):e0173670. doi: 10.1371/journal.pone.0173670 (PMC5345865; doi:10.1371/journal.pone.0173670)
Supplement: S2 Table — Dates in parentheses are rejected due to extreme age reversal. (PDF) [file pone.0173670.s003.pdf]

Core ID	Depth (cm)	Material	Conventional C^{14} age (yr BP)	2-σ Calibrated range (Cal yr BP)
**SRM**	56	Leaf	145 ±25	0 - 280
	139	Leaf	1180 ±30	990 - 1180
	179	Leaf	1940 ±30	1820 – 1970
	243	leaf	2860 ±30	2880 – 3070
	246	Wood	155 ±25	(Rejected)
	246	Wood	> Modern	(Rejected)
	260	Leaf	2240 ±30	(2150 – 2340)
	300	Bark	3540 ±35	3700 – 3910
	374	Organic silt	4160 ±30	4780 - 4830
	374	Roots	2940 ±30	(3000 – 3210)
	440	Leaf	1090 ±20	(Rejected)
	446	Organic silt	5800 ±30	6500 - 6670
	446	Roots	4060 ±30	(4760 – 4800)
	448	Plant debris	6620 ±260	(6940 – 7980)
**SRS-6**	114	Leaf	500 ±25	510 – 540
	200	Organic silt	2260 ±20	2160-2340
	232	Leaf	2970 ±90	2920-3360
	303	Organic silt	3570 ±25	3770-3970
	312	Leaf	1680 ±25	(1530-1630)
	378	Organic silt	4230 ±40	4630-4860
	381	Leaf	2580 ±40	(2500-2770)
**SRS-5**	80	Leaf	930 ±25	790-920
	155	Organic silt	2050 ±25	1930-2110
	171	Leaf	1270 ±25	(1150-1280)
	190	Organic silt	2560 ±20	2540-2750
	240	Leaf	1280 ±30	(1150-1290)
	248	Organic silt	4010 ±25	4420-4520
**SRS-4**	42	Leaf	> Modern	(Rejected)
	50	Leaf	145 ±20	0-280
	90	Organic silt	1180 ±30	990-1220
	100	Organic silt	1530 ±20	1360-1520
	125	Organic silt	2460 ±20	2400-2700
	151	Leaf	2330 ±50	(2160-2680)
	156	Shell hashes	3900 ±25	4250-4420

Table S2. Radiocarbon dating results for core SRM, SRS-6, SRS-5, and SRS-4. Dates in parentheses are rejected due to extreme age reversal.
